# Solanidine is a sensitive and specific dietary biomarker for CYP2D6 activity

**DOI:** 10.1186/s40246-024-00579-8

**Published:** 2024-02-01

**Authors:** Johanna I. Kiiski, Mikko Neuvonen, Mika Kurkela, Päivi Hirvensalo, Kreetta Hämäläinen, E. Katriina Tarkiainen, Johanna Sistonen, Mari Korhonen, Sofia Khan, Arto Orpana, Anne M. Filppula, Marko Lehtonen, Mikko Niemi

**Affiliations:** 1 Individualized Drug Therapy Research Program, Faculty of Medicine, University of Helsinki, Helsinki, Finland; 2 Department of Clinical Pharmacology, University of Helsinki, Helsinki, Finland; 3 Department of Clinical Pharmacology, HUS Diagnostic Center, Helsinki University Hospital, Helsinki, Finland; 4 Genetics Laboratory, HUS Diagnostic Center, Helsinki University Hospital, Helsinki, Finland; 5 Pharmaceutical Sciences Laboratory, Åbo Akademi University, Turku, Finland; 6 School of Pharmacy, University of Eastern Finland, Kuopio, Finland

**Keywords:** CYP2D6, Solanidine, Drug metabolism, Biomarkers

## Abstract

**Background:**

Individual assessment of CYP enzyme activities can be challenging. Recently, the potato alkaloid solanidine was suggested as a biomarker for CYP2D6 activity. Here, we aimed to characterize the sensitivity and specificity of solanidine as a CYP2D6 biomarker among Finnish volunteers with known *CYP2D6* genotypes.

**Results:**

Using non-targeted metabolomics analysis, we identified 9152 metabolite features in the fasting plasma samples of 356 healthy volunteers. Machine learning models suggested strong association between *CYP2D6* genotype-based phenotype classes with a metabolite feature identified as solanidine. Plasma solanidine concentration was 1887% higher in genetically poor CYP2D6 metabolizers (gPM) (*n* = 9; 95% confidence interval 755%, 4515%; *P* = 1.88 × 10^–11^), 74% higher in intermediate CYP2D6 metabolizers (gIM) (*n* = 89; 27%, 138%; *P* = 6.40 × 10^–4^), and 35% lower in ultrarapid CYP2D6 metabolizers (gUM) (*n* = 20; 64%, − 17%; *P* = 0.151) than in genetically normal CYP2D6 metabolizers (gNM; *n* = 196). The solanidine metabolites *m/z* 444 and 430 to solanidine concentration ratios showed even stronger associations with CYP2D6 phenotypes. Furthermore, the areas under the receiver operating characteristic and precision–recall curves for these metabolic ratios showed equal or better performances for identifying the gPM, gIM, and gUM phenotype groups than the other metabolites, their ratios to solanidine, or solanidine alone. In vitro studies with human recombinant CYP enzymes showed that solanidine was metabolized mainly by CYP2D6, with a minor contribution from CYP3A4/5. In human liver microsomes, the CYP2D6 inhibitor paroxetine nearly completely (95%) inhibited the metabolism of solanidine. In a genome-wide association study, several variants near the *CYP2D6* gene associated with plasma solanidine metabolite ratios.

**Conclusions:**

These results are in line with earlier studies and further indicate that solanidine and its metabolites are sensitive and specific biomarkers for measuring CYP2D6 activity. Since potato consumption is common worldwide, this biomarker could be useful for evaluating CYP2D6-mediated drug–drug interactions and to improve prediction of CYP2D6 activity in addition to genotyping.

**Supplementary Information:**

The online version contains supplementary material available at 10.1186/s40246-024-00579-8.

## Background

*CYP2D6* is one of the most variable genes from the cytochrome P450 (*CYP*) family, characterized by more than one hundred known sequence and copy number variations (deletions and duplications), as well as hybrid alleles containing sequences from the neighboring *CYP2D7* pseudogene. This variation affects widely the pharmacokinetics, efficacy, and safety of common medications, including many antidepressants, antipsychotics, analgesics, cardiovascular, and anticancer drugs metabolized via CYP2D6 [1–3].

The broad genetic variation of *CYP2D6* leads to significant differences in individual metabolic capacities ranging from poor to ultrarapid metabolism [2]. Genotyping the *CYP2D6* variants does not, however, completely predict CYP2D6 activity, because multiple non-genetic factors also play a role. These factors include, for example, epigenetic regulation, dietary compounds, pregnancy, and especially drug–drug interactions (DDI). Furthermore, several *CYP2D6* variants are not yet assigned with a metabolic function [4, 5]. Therefore, identifying for example dietary probe substrates as biomarkers of CYP2D6 activity would be beneficial for evaluating CYP2D6-mediated DDIs and to further improve the prediction of enzyme activity in addition to genotyping [6–8].

A biomarker present in human urine named M1 (ion *m/z* 444.3102) was found in a non-targeted metabolomics study in 2014 [9] and was later identified as a metabolite of the potato alkaloid solanidine, based on similar fragmentation pattern in mass spectrometry [10]. The compound was absent in the urine of CYP2D6 poor metabolizers and its formation was reduced when CYP2D6 specific inhibitors were used [9, 10]. Recently, ion *m/z* 444.3102 was further isolated, purified, and determined to be 3,4-seco-solanidine-3,4-dioic acid (SSDA) by using chromatographic separation techniques and mass spectrometry analysis [11]. Other studies further suggested M414 metabolite-to-solanidine-ratios could predict CYP2D6 poor metabolizer phenotype [12] and showed association between solanidine metabolism and CYP2D6-mediated risperidone metabolism [13].

These data suggest that solanidine and its metabolites could serve as dietary biomarkers for CYP2D6 activity. However, the sensitivities and specificities of solanidine and its metabolites as CYP2D6 biomarkers require further investigation, in particular in intermediate and ultrarapid CYP2D6 metabolizers. Therefore, our aim was to identify possible other CYP2D6 biomarkers present in human plasma by non-targeted metabolomics analysis of a large sample set of *CYP2D6* genotyped healthy Finnish volunteers with a high frequency of genetically ultrarapid CYP2D6 metabolizers [14–16], and to further examine the specificity and sensitivity of solanidine as a biomarker of CYP2D6 activity. Hence, we employed *CYP2D6* genotypes as a proxy for CYP2D6 activity and genome-wide analysis to identify possible other genes associated with solanidine and its metabolite levels.

## Methods

### Subjects

A total of 356 DNA and plasma samples from unrelated healthy Finnish volunteers (183 women, 173 men) from previous pharmacogenetics studies were available for analysis. All participants were non-smokers with no concomitant medication. The mean ± standard deviation (SD) age of the participants was 24.1 ± 4.1 years, weight 69.7 ± 12.1 kg and body mass index 22.9 ± 2.7 kg/m^2^ (Additional file 1: Table S1). Blood samples were collected after overnight fasting during two periods (2012–2014 and 2015–2017) and stored in − 70 °C or − 80 °C as previously described [17–20]. Microarray-based genome-wide genotyping data for the participants was obtained from a previously published study. [18]

### *CYP2D6* and *NFIB* genotyping and genotype-to-phenotype translations

Genomic DNA was extracted from ethylenediaminetetraacetic acid (EDTA) blood samples with the Maxwell 16 LEV Blood DNA Kit on a Maxwell 16 Research automated nucleic acid extraction system (Promega, Madison, WI). DNA samples were genotyped for *CYP2D6* copy number variations and 19 clinically relevant *CYP2D6* sequence variants defining the *2, *3, *4, *5, *6, *9, *10, *11, *13, *17, *29, *39, *41, *59, *65, *69, and *88 alleles, as named in the Pharmacogene Variation (PharmVar) Consortium *CYP2D6* core allele definitions. Alleles without the studied variants were named as *1. The detailed information of each variant allele is listed in Additional file 1: Table S2 [2, 12, 22]. Genotyping was performed with a clinical pharmacogenetic panel test available at the Genetics Laboratory of the HUS Diagnostic Center (Helsinki University Hospital, Helsinki, Finland) using massive parallel sequencing on the Ion GeneStudio™ S5 Prime System (Thermo Fisher Scientific, Waltham, MA) as previously described [23]. Allele-specific *CYP2D6* copy number was inferred from variant allele frequencies, which were verified in a subset of samples using TaqMan^®^-assays (Thermo Fisher Scientific) targeting *2- (C__27102425_50), *4- (C__27102431_D0), and *41-specific variants (C__34816116_20). Assays were run on the Droplet Digital PCR System (Bio-Rad Laboratories, Hercules, CA) following manufacturer’s instructions.

To classify the expected metabolic rate of each study subject into phenotype, the activity score system (AS), standardized by the Clinical Pharmacogenetics Implementation Consortium (CPIC) and Dutch Pharmacogenetics Working Group, was utilized [24, 25]. Each allele was assigned to value of 0 (no function), 0.25–0.5 (decreased), or 1 (normal function). The sum of the values of each allele was used to determine the metabolizer phenotypes and predict the activity of the CYP2D6 enzyme (Additional file 1: Table S3). The AS of 0 equals to genetically poor metabolizer (gPM), AS 0 < *x* < 1.25 to intermediate metabolizer (gIM), AS 1.25 ≤ *x* ≤ 2.25 to normal metabolizer (gNM,) and AS > 2.25 to ultrarapid metabolizer (gUM). [26–29]

CYP2D6 activity-influencing variant *NFIB* rs28379954 was genotyped with a functionally tested TaqMan^®^-assay using QuantStudio 12 K Flex real-time PCR equipment following manufacturer’s instructions (ThermoFisher Scientific).

### Plasma metabolite isolation and non-targeted metabolite profiling

Plasma sample preparation for metabolite analyses was done as described previously [30]. Briefly, samples were thawed on ice and a 100 µL aliquot of plasma was dispensed into a 96-well filter plate (Captiva ND, 0.2 mm PP, Agilent Technologies, Waldbronn, Germany) containing 400 µL of ice-cold acetonitrile. Samples were mixed to thoroughly precipitate plasma proteins, and then centrifuged 700×*g* for 5 min at 4 °C and the supernatants were collected to a 96-well storage plate and stored at 10 °C.

Non-targeted metabolic profiling was performed at the LC–MS metabolomics center of Biocenter Kuopio (University of Eastern Finland, Finland). The analysis was carried out using an ultra-high-performance liquid chromatography (Vanquish Flex UHPLC system, Thermo Scientific, Bremen, Germany) coupled online to a high-resolution mass spectrometry (Q Exactive Focus, Thermo Scientific). All samples were analyzed using two different chromatographic techniques, i.e., reversed phase (RP) and hydrophilic interaction chromatography (HILIC). Data were acquired in both electrospray ionization (ESI) polarities, i.e., ESI positive (ESI+) and ESI negative (ESI−). Data-dependent product ion spectrums (MS2 data) were acquired from pooled quality control (QC) samples at the beginning and end of the analysis for each mode. QC samples were injected in the beginning of the analysis and after every 12 samples. The LC–MS instrument setups and data acquisition parameters have been described previously [31].

### In vitro studies

In vitro studies were carried out to characterize the roles of drug-metabolizing enzymes in solanidine metabolism. First, the formation of hydroxy (OH) solanidine (*m*/*z* 414) was screened in 11 human recombinant CYP enzymes. Thereafter, the depletion of solanidine and formation of *m*/*z* 414 was investigated in human liver microsomes (HLMs) and in human recombinant CYP2D6. The HLM incubations were performed with and without the CYP2D6-specific inhibitor paroxetine and the CYP3A4/5-specific inhibitor ritonavir [32, 33]. Moreover, the formation of glucuronide and sulfate metabolites was investigated in HLMs and human liver cytosol (HLC), respectively. Longer incubations containing higher concentrations of HLM protein and solanidine were carried out to investigate the formation of metabolites other than *m*/*z* 414. Detailed descriptions of the in vitro methods are presented in the Supplementary Material S1.

### Quantification of solanidine and its metabolites

Solanidine calibration samples (0–25 ng/mL) and quality control samples were prepared in charcoal-stripped plasma using a commercial reference standard (Toronto Research Chemicals, North York, ON). Prior to quantification, 100 µL plasma samples were precipitated with 300 µL of acetonitrile containing the internal standard (IS), and the supernatants were purified using an Impact protein precipitation plate (Phenomenex, Torrance, CA). Supernatants were then dried in a centrifugal evaporator (Genevac, Thermo Fisher Scientific, Waltham, MA) and reconstituted in 50 µL of 80% methanol. The chromatographic separation of solanidine and its metabolites was achieved on Kinetex C18 analytical column (2.1 × 75 mm internal diameter, 2.6 µm particle size) (Phenomenex, Torrance, CA) using gradient elution. The gradient of mobile phase was a mixture of 0.1% formic acid (mobile phase A) and acetonitrile (mobile phase B) as follows: 0.5 min at 25% B on hold, a linear ramp from 10 B to 90% B over 7.2 min, 1.8 min at 90% B on hold followed by equilibration back to 10% B. Mobile phase was delivered at 300 µL/min and the oven temperature was set at 40 °C. The ABSciex 6500 Qtrap mass spectrometer (Toronto, ON) was operated in positive multiple reaction monitoring (MRM) mode using the protonated precursor ions and the characteristic *m*/*z* 98 product ion for solanidine and its metabolites [10]. Data were analyzed using Analyst software version 1.6.3 (ABSciex, Toronto, ON) using linear regression applying 1/concentration weighting and a peak area ratio between the analyte and IS. Isotope labeled tauroursodeoxycholate (TUDCA) TUDCA-D5, served as IS for solanidine and for all the metabolites monitored. The lower limit of quantification (LLOQ) for solanidine was 0.01 ng/mL, and the between-day (*n* = 6) coefficients of variation (CV%) of quality controls at concentration levels 0.05, 1.0 and 10 ng/mL were 10%, 6.5% and 9.9%, respectively. Solanidine showed linear response (*r* > 0.995) over the concentration range from 0.01 to 25 ng/mL. No authentic reference standards were commercially available for solanidine metabolites. Semi-quantitative concentrations of solanidine metabolites are given in arbitrary units based on the responses of metabolite peak area/IS area ratios. For each metabolite, a signal-to-noise (*S*/*N*) ratio of 10 was defined as the lowest measurable level, which approximately corresponded to the solanidine *S*/*N* ratio at LLOQ level. The response linearities of solanidine metabolites (*r* > 0.999) were measured from an *S*/*N* ratio of 10 using dilution series of study samples. The same analytical system was used for the measurement of in vitro samples.

### Statistical analyses

The non-targeted metabolomics molecular feature data were normalized by removing the median and scaling the data to the interquartile range using the robust scaler procedure from sklearn-library [34]. Two decision tree-based machine learning algorithms, random forests (RFs) and gradient boosted decision trees (GBDTs) were employed in parallel to identify metabolite features associated with the CYP2D6 phenotype classes [35, 36]. The RFs and GBDTs were implemented as regressors operating with continuous values with Python version 3.8.3 using sklearn-library [34]. Normalized metabolite molecular feature data were used as the input and numeric CYP2D6 phenotype classes as target values. The optimal hyperparameters were searched by sampling from specified ranges and using randomized search cross-validation for both RFs and GBDTs. To minimize overfitting, cross-validation was carried out with 5*twofold nested cross-validation. The strength of association of each metabolite feature with the CYP2D6 phenotype was quantified by calculating the average decrease in Gini impurity, a measurement of likelihood of an incorrect classification of a variable. A Gini impurity decrease of > 0.01 was considered as a significant contribution to phenotype classification.

The plasma solanidine and its metabolite data were analyzed with the statistical programs JMP Genomics 8.0 (SAS Institute Inc., Cary, NC) and IBM SPSS 25 for Windows (Armonk, NY). The concentrations of solanidine and its metabolites were logarithmically transformed before analysis. Subjects with plasma solanidine concentrations lower than the LLOQ (0.01 ng/mL) were excluded from the analysis. In addition, solanidine metabolite levels below the LLOQ (signal-to-noise ratio < 10) were set to half of the LLOQs. Possible differences in solanidine and its metabolite concentrations between the two clinical trials in which the samples were collected, and effects of demographic covariates (sex and logarithmically transformed bodyweight) were investigated using a forward stepwise linear regression analysis. *P*-value thresholds of 0.05 for entry and 0.10 for removal were employed as the stepping method criteria.

Genome-wide association analyses for solanidine concentration and metabolite to solanidine ratios were performed using linear regression analysis with additive coding and the significant demographic covariates set as fixed factors. Logarithmically transformed solanidine concentration and metabolite to solanidine ratios were treated as continuous variables. A *P*-value of below 5 × 10^–8^ was considered genome wide significant. For genotype-predicted CYP2D6 phenotype classes, analysis of variance adjusting for significant demographic covariates was carried out with pairwise comparisons with the Fisher’s least significant difference method. A *P*-value of below 0.05 was considered statistically significant. Precision–recall (PR) and receiver operating characteristic (ROC) analyses were carried out and areas under the precision–recall (AUPRC) and receiver operating characteristic (AUROC) curves were calculated with MedCalc Statistical Software v.19.7 (MedCalc Software bv, Ostend, Belgium) using 1000 bootstrap iterations for confidence intervals.

## Results

**Fig. 1:**
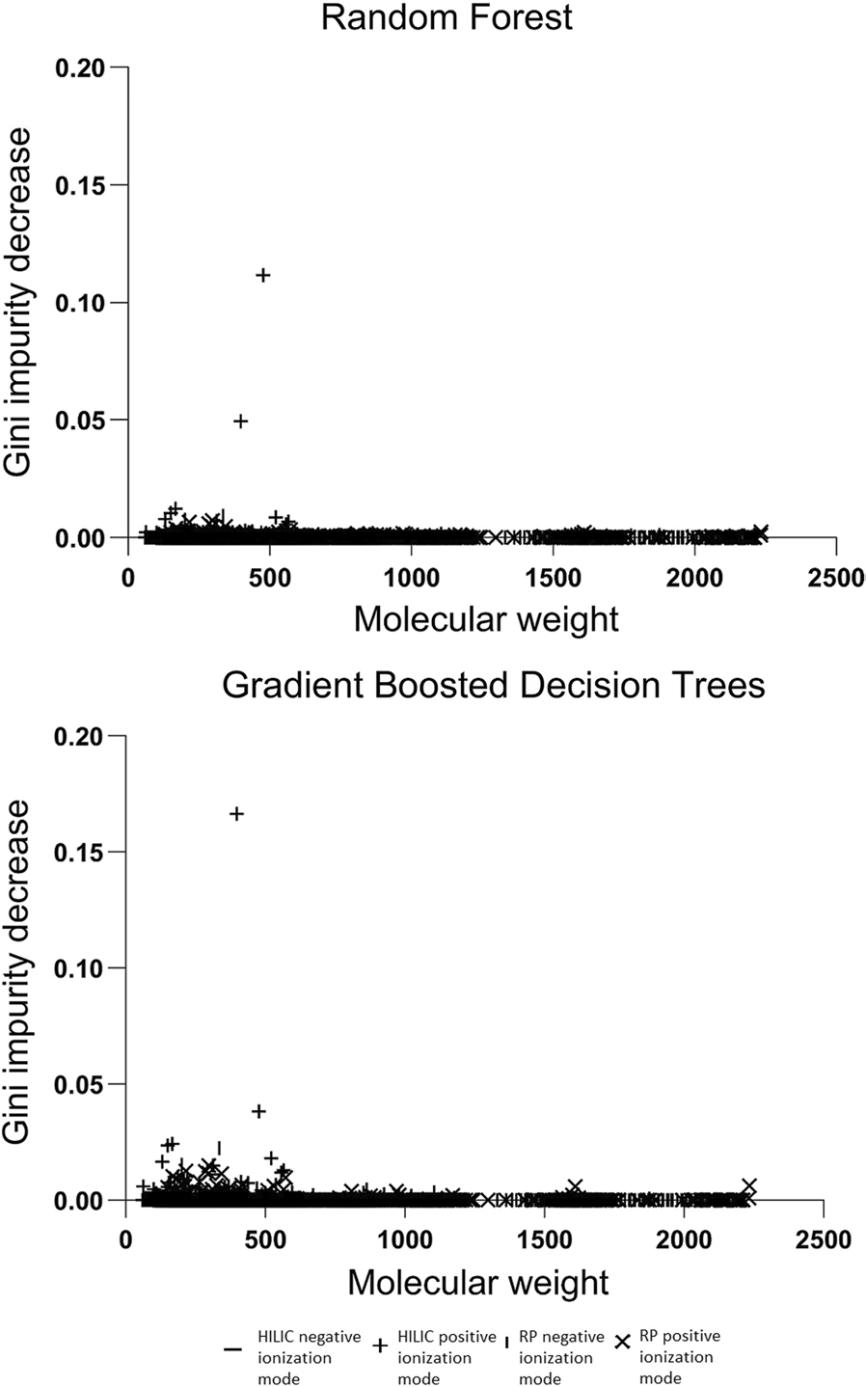
Associations of metabolite features with the CYP2D6 phenotype classes in the machine earning algorithms

A total of 9152 metabolite features were found in the non-targeted metabolome analysis of the plasma samples. The machine learning models showed the strongest CYP2D6 phenotype class association with a feature with a molecular weight of 397.33 in the positive ionization mode and a retention time of 0.53 min in the HILIC chromatography, as indicated by a Gini impurity decrease of 0.166 in GBDT (Fig. 1, Additional file 1: Table S4). This metabolite was preliminarily identified as solanidine and the finding was confirmed using pure reference compound (metabolite identification level LI 1). In addition, a relatively strong association was seen with a feature with a molecular weight of 477.29 in the positive ionization mode and a retention time of 0.65 min in the HILIC chromatography. This feature showed mass fragmentation spectra characteristic of a phosphatidylethanolamine group lipid, but the compound could not be identified further (LI 3).

### Effect of CYP2D6 phenotype on solanidine and solanidine metabolite concentrations

Of the 356 healthy volunteers, one subject was excluded because the CYP2D6 phenotype could not be inferred from the genotype (Additional file 1: Table S3). Moreover, 41 subjects (one CYP2D6 gPM, five gIM, 34 gNM, and one gUM phenotype) were excluded because their plasma solanidine levels were below the LLOQ. Within the final study group of 314 subjects, in addition to solanidine, measurable amounts of solanidine metabolite ions *m*/*z* 414, 416, 444, 430, and 412 were found in 310, 308, 299, 296, and 225 subjects, respectively.

**Fig. 2:**
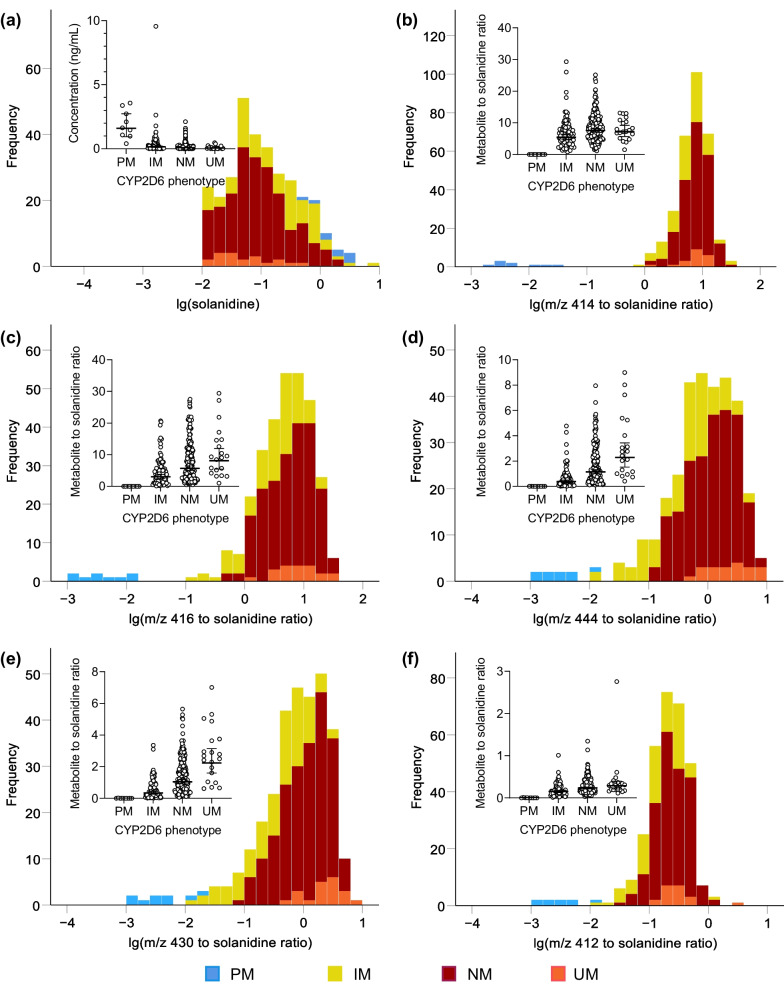
Frequency distribution histograms of log solanidine concentrations (**a**) and log metabolic ratios (**b**–**f**). CYP2D6 gPM, gIM, gNM, and gUM phenotypes are depicted by colors. Insets show individual solanidine concentrations and metabolite to solanidine concentration ratios in CYP2D6 gPM, gIM, gNM, and gUM individuals (horizontal line, geometric mean; whiskers, 95% confidence intervals). gIM, intermediate metabolizer; gNM, normal metabolizer; gPM, poor metabolizer; gUM, ultrarapid metabolizer

The geometric mean (95% CI; range) of fasting plasma solanidine concentration was 0.110 (0.095, 0.128; 0.01–9.55) ng/mL. The concentrations were 1887% higher in the gPM group (*n* = 9; 95% CI 755%, 4515%; *P* = 1.88 × 10^–11^), 73.7% higher in the gIM group (*n* = 89; 26.7%, 138%; *P* = 6.40 × 10^–4^), and 34.7% lower in the gUM group (*n* = 20; 63.6%, − 17.0%; *P* = 0.151) than in the gNM group (*n* = 196) (Table 1, Fig. 2). Accordingly, the lowest levels of solanidine metabolites, *m*/*z* 414, 416, 444, 430, and 412 were found among gPM subjects. The genotype-predicted phenotype classes showed strong associations with plasma metabolite to solanidine ratios (Table 1). Particularly the *m*/*z* 444 and 430 to solanidine ratios showed significant associations with the CYP2D6 gPM, gIM, and gUM phenotypes. The ion *m/z* 444 to solanidine ratio was 99.7% lower in the gPM (95% CI, 99.9%, 99.4%;* P* = 5.23 × 10^–42^), 66.4% lower in gIM (74.4%, 56.0%; *P* = 4.46 × 10^–14^) and 101% higher in the gUM group (21.3%, 232%; *P* = 0.00682) than in the gNM group. Similarly, *m/z* 430 to solanidine ratio was 99.6% lower in the gPM (99.8%, 99.2%, *P* = 3.48 × 10^–42^), 66.5% lower in the gIM (74.0%, 56.8%; *P* = 9.73 × 10^–16^), and 108% higher in the gUM (30.1%, 233%; *P* = 0.00234) than in the gNM group (Table 1, Additional file 1: Table S5, Fig. 2).

**Table 1:** Effect of CYP2D6 phenotype on plasma solanidine and metabolite to solanidine ratios in healthy volunteers (*n* = 314)

Phenotype (n)	Geometric mean (95% CI)	Ratio to normal function (95% CI)	*P*
*Feature: Solanidine (ng/mL)*			
Poor metabolizer (9)	1.76 (0.775, 4.02)	19.9 (8.55, 46.1)	1.88 × 10^–11^
Intermediate metabolizer (89)	0.154 (0.119, 0.200)	1.74 (1.27, 2.38)	6.40 × 10^–4^
Normal metabolizer (196)	0.0889 (0.0746, 0.106)	1.00	
Ultrarapid metabolizer (20)	0.0580 (0.0333, 0.101)	0.653 (0.364, 1.17)	0.151
*Feature: m/z 414 to solanidine peak area ratio*			
Poor metabolizer (9)	0.00649 (0.00441, 0.00954)	0.000863 (0.000582, 0.00128)	1.31 × 10^–109^
Intermediate metabolizer (89)	5.49 (4.86, 6.20)	0.731 (0.630, 0.845)	3.75 × 10^–5^
Normal metabolizer (196)	7.52 (6.92, 8.16)	1.00	
Ultrarapid metabolizer (20)	7.20 (5.55, 9.34)	0.958 (0.729, 1.26)	0.758
*Feature: m/z 416 to solanidine peak area ratio*			
Poor metabolizer (9)	0.00433 (0.00231, 0.00811)	0.000765 (0.000402, 0.00146)	6.84 × 10^–65^
Intermediate metabolizer (89)	3.08 (2.53, 3.76)	0.545 (0.429, 0.693)	1.11 × 10^–6^
Normal metabolizer (196)	5.65 (4.95, 6.47)	1.00	
Ultrarapid metabolizer (20)	7.99 (5.23, 12.2)	1.41 (0.905, 2.20)	0.128
*Feature: m/z 444 to solanidine peak area ratio*			
Poor metabolizer (9)	0.00319 (0.00157, 0.00648)	0.00280 (0.00135, 0.00579)	5.23 × 10^–42^
Intermediate metabolizer (89)	0.382 (0.305, 0.479)	0.335 (0.256, 0.440)	4.46 × 10^–14^
Normal metabolizer (196)	1.14 (0.979, 1.33)	1.00	
Ultrarapid metabolizer (20)	2.29 (1.42, 3.69)	2.01 (1.21, 3.32)	0.00682
*Feature: m/z 430 to solanidine peak area ratio*			
Poor metabolizer (9)	0.00420 (0.00217, 0.00816)	0.00406 (0.00206, 0.00801)	3.48 × 10^–42^
Intermediate metabolizer (89)	0.347 (0.281, 0.428)	0.335 (0.260, 0.432)	9.73 × 10^–16^
Normal metabolizer (196)	1.04 (0.899, 1.19)	1.00	
Ultrarapid metabolizer (20)	2.15 (1.38, 3.37)	2.08 (1.30, 3.33)	0.00234
*Feature: m/z 412 to solanidine peak area ratio*			
Poor metabolizer (9)	0.00331 (0.00208, 0.00527)	0.0140 (0.00869, 0.0225)	1.22 × 10^–48^
Intermediate metabolizer (89)	0.152 (0.131, 0.177)	0.644 (0.539, 0.769)	1.80 × 10^–6^
Normal metabolizer (196)	0.236 (0.214, 0.261)	1.00	
Ultrarapid metabolizer (20)	0.345 (0.252, 0.473)	1.460 (1.05, 2.03)	0.0245

A total of 17 individuals with the *CYP2D6* gNM genotype were carriers of *NFIB* rs28379954 variant, which has previously been suggested to convert normal CYP2D6 metabolizers to ultrarapid metabolizers [37, 38]. Moving these subjects into the gUM group weakened the associations (Additional file 1: Table S6).

### Performance of solanidine and its metabolites as CYP2D6 biomarkers

**Fig. 3:**
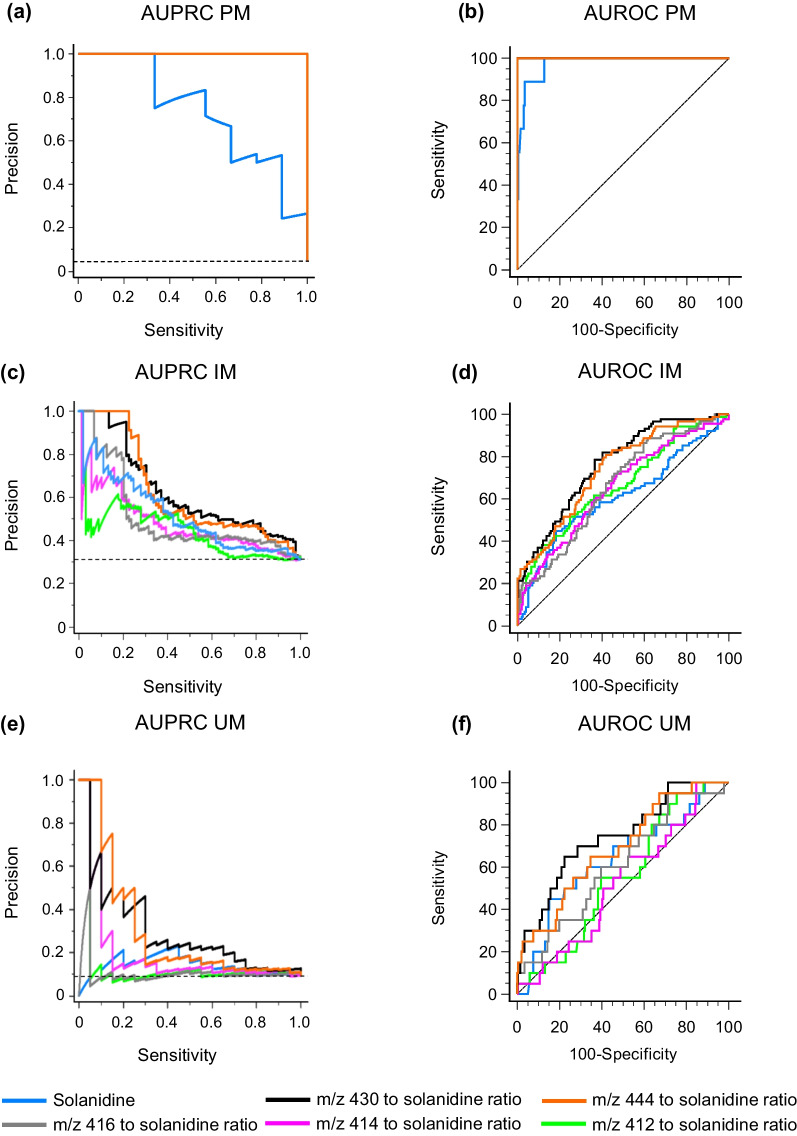
Performance of plasma biomarkers in detecting different CYP2D6 phenotypes. AUPRCs represent the trade-off of precision and sensitivity for every possible cutoff value. Horizontal dashed lines (**a**, **c**, **e**) represent the baseline AUPRC values, which correspond to the proportions of gPM, gIM, and gUM phenotypes. AUROCs show relationships between sensitivity and specificity for every possible cutoff value. Dashed lines (**b**, **d**, **f**) represent random classifier. AUPRC, area under the precision–recall curve; AUROC, area under the receiver operating characteristic curve; gIM, intermediate metabolizer; gPM, poor metabolizer; gUM ultrarapid metabolizer

To investigate the performances of solanidine and the metabolite to solanidine ratios as CYP2D6 biomarkers, we calculated the AUROC and AUPRC values for these features (Fig. 3, Table 2). AUROC (0.976) and AUPRC (0.730) for the gPM versus gNM group demonstrated rather good classification potential for the plasma solanidine alone, but all the metabolite to solanidine ratios behaved even better, being all perfect classifiers (AUROCs and AUPRCs 1.0). In comparison with solanidine, all the metabolite to solanidine ratios discriminated gPM from the gNM group with significantly higher AUPRC values than solanidine alone, but the differences in AUROC values were not statistically significant. Regarding the gIM group, the *m/z* 444 and 430 to solanidine ratios showed statistically significantly better performances compared to solanidine using both the AUPRC and AUROC metrics, whereas the *m/z* 414, 416, and 412 to solanidine ratios performed similarly to solanidine. Regarding the gUM group, none of the metabolite to solanidine ratios showed statistically significantly better performances than solanidine*.*

**Table 2:** Comparison of plasma biomarker performances in detecting different CYP2D6 phenotypes versus normal metabolizers (*n* = 196)

CYP2D6 biomarker	Mean AUPRC (95% CI)	F1_max_	Mean difference from solanidine (95% CI)	Mean AUROC (95% CI)	Difference from solanidine	
					Mean (95% CI)	*P* value
*Poor metabolizer (n = 9) baseline AUPRC = 0.0439 and baseline AUROC = 0.5*						
Solanidine	0.730 (0.383, 0.922)	0.667		0.976 (0.944, 0.992)		
*m*/*z* 414 to solanidine ratio	1.000	1.000	0.270 (0.0853, 0.583)^*^	1.000 (0.982, 1.000)	0.0241 (− 0.00503, 0.0532)	0.105
*m*/*z* 416 to solanidine ratio	1.000	1.000	0.270 (0.0853, 0.583)^*^	1.000 (0.982, 1.000)	0.0241 (− 0.00503, 0.0532)	0.105
*m*/*z* 444 to solanidine ratio	1.000	1.000	0.270 (0.0853, 0.583)^*^	1.000 (0.982, 1.000)	0.0241 (− 0.00503, 0.0532)	0.105
*m*/*z* 430 to solanidine ratio	1.000	1.000	0.270 (0.0853, 0.583)^*^	1.000 (0.982, 1.000)	0.0241 (− 0.00503, 0.0532)	0.105
*m*/*z* 412 to solanidine ratio	1.000	1.000	0.270 (0.0853, 0.583)^*^	1.000 (0.982, 1.000)	0.0241 (− 0.00503, 0.0532)	0.105
*Intermediate metabolizer (n = 89) baseline AUPRC = 0.312 and baseline AUROC = 0.5*						
Solanidine	0.444 (0.344, 0.548)	0.488		0.610 (0.550, 0.667)		
*m*/*z* 414 to solanidine ratio	0.477 (0.376, 0.580)	0.522	0.0333 (-0.123, 0.185)	0.652 (0.594, 0.707)	0.0423 (− 0.0387, 0.123)	0.306
*m*/*z* 416 to solanidine ratio	0.504 (0.402, 0.606)	0.547	0.0604 (-0.0909, 0.204)	0.662 (0.604, 0.717)	0.0525 (− 0.0131, 0.118)	0.117
*m*/*z* 444 to solanidine ratio	0.620 (0.515, 0.714)	0.593	0.176 (0.0263, 0.316)^*^	0.741 (0.686, 0,791)	0.131 (0.0729, 0.189)	< 0.0001^*^
*m*/*z* 430 to solanidine ratio	0.628 (0.523, 0.722)	0.606	0.184 (0.0321, 0.327)^*^	0.764 (0.710, 0.812)	0.154 (0.0903, 0.218)	< 0.0001^*^
*m*/*z* 412 to solanidine ratio	0.519 (0.416, 0.621)	0.524	0.0753 (− 0.0872, 0.226)	0.666 (0.608, 0.720)	0.0562 (− 0.0171, 0.129)	0.133
*Ultrarapid metabolizer (n = 20) baseline AUPRC = 0.0926 and baseline AUROC = 0.5*						
Solanidine	0.139 (0.0434, 0.364)	0.305		0.636 (0.568, 0.701)		
*m*/*z* 414 to solanidine ratio	0.104 (0.0271, 0.328)	0.194	− 0.0343 (0.0258, − 0.105)	0.524 (0.455, 0.592)	− 0.112 (0.0913, − 0.316)	0.280
*m*/*z* 416 to solanidine ratio	0.195 (0.0744, 0.423)	0.218	0.0566 (− 0.0836, 0.266)	0.610 (0.542, 0.676)	− 0.0263 (0.108, − 0.161)	0.702
*m*/*z* 444 to solanidine ratio	0.295 (0.138, 0.522)	0.333	0.156 (− 0.0345, 0.391)	0.689 (0.622, 0.750)	0.0520 (− 0.0700, 0.174)	0.403
*m*/*z* 430 to solanidine ratio	0.296 (0.138, 0.523)	0.364	0.157 (− 0.0306, 0.400)	0.741 (0.678, 0.798)	0.105 (− 0.0384, 0.248)	0.152
*m*/*z* 412 to solanidine ratio	0.144 (0.0462, 0.370)	0.203	0.00553 (− 0.0969, 0.216)	0.551 (0.482, 0.619)	− 0.0851 (0.0731, − 0.243)	0.292

AUPRC, area under precision–recall curve; AUROC, area under receiver operating characteristic curve; CI, confidence interval; F1_max_, harmonic mean of the precision and recall over all measurement levels. ^*^Statistically significant difference

### Genome-wide association study for plasma solanidine, and metabolite to solanidine ratios

**Fig. 4:**
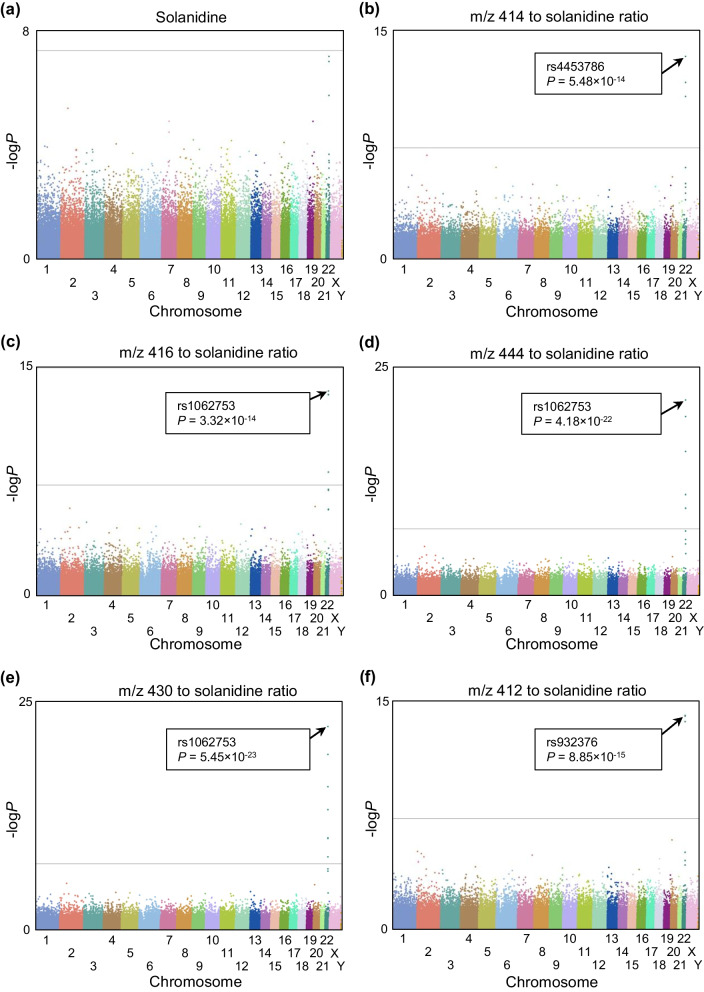
Manhattan plots of the fasting plasma (**a**) solanidine and (**b**–**f**) metabolite to solanidine ratios. Horizontal lines represent the genome-wide significance level

In a genome-wide association analysis, the strongest associations with the plasma metabolite to solanidine ratios were found between *SEPTIN3* 3’UTR variants rs1062753 and *m/z* 430 (*P* = 5.45 × 10^–23^), and rs1062753 and *m/z* 444 (*P* = 4.18 × 10^–22^) (Fig. 4). In addition, the variant also associated with the *m/z* 416 to solanidine ratio. Two intronic *TCF20* variants, rs4453786 and rs932376, associated with the *m/z* 414 and *m/z* 412 to solanidine ratios. When adjusting for the effect of the single nucleotide variations (SNVs) with the strongest associations, the remaining genome-wide associations, if any, were close to borderline significance, indicating strong linkage disequilibrium between these SNVs. No SNV was genome-wide significantly associated with parent solanidine levels. Both genes are located near *CYP2D6* on the chromosome 22*.* The *SEPTIN3* rs1062753, and the *TCF20* rs4453786 and rs932376 variants showed a strong linkage disequilibrium with the *CYP2D6* rs3892097 variant defining the CYP2D6*4 allele (*r*^2^ = 0.68, *P* = 1.18 × 10^–51^, *r*^2^ = 0.84, *P* = 4.39 × 10^–67^, and *r*^2^ = 0.58, *P* = 2.12 × 10^–46^, respectively).

### In vitro experiments

**Fig. 5:**
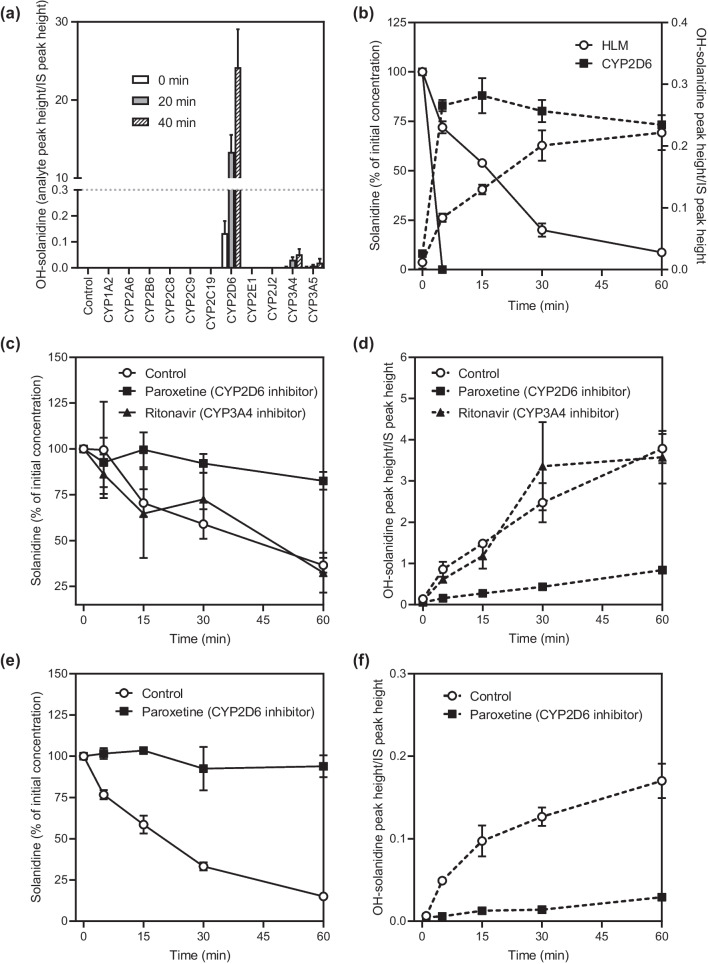
In vitro investigations of solanidine metabolism. Formation of OH-solanidine (m/z 414) in 11 recombinant CYP enzyme and control bactosome incubations (0.1 mg/mL) from solanidine 2 µM (**a**); Depletion of 10 nM solanidine and formation of OH-solanidine in HLM (0.2 mg/mL) and CYP2D6 (0.1 mg/mL) incubations (**b**); Effects of the CYP2D6 inhibitor paroxetine and CYP3A inhibitor ritonavir on the depletion of 0.1 µM solanidine (**c**) and subsequent formation of OH–solanidine (**d**) in HLMs (0.2 mg/mL); Effects of paroxetine on the depletion of 10 nM solanidine (**e**) and subsequent formation of OH-solanidine in HLMs (0.2 mg/mL) (**f**). CYP, cytochrome P450; HLMs, human liver microsomes

Among the 11 screened human recombinant CYP enzymes, CYP2D6 was clearly the major enzyme involved in the formation of OH-solanidine (*m/z* 414) (Fig. 5a). Some OH-solanidine was detected also in CYP3A4/5 incubations, but the amount was very low compared to that in CYP2D6 incubations.

Incubation of a clinically relevant concentration of solanidine (10 nM) with CYP2D6 resulted in a complete depletion of solanidine already at 5 min (< 1% of the initial concentration remaining in the incubation) (Fig. 5b). With HLMs, the depletion of solanidine was slower and approximately 75% of the initial substrate concentration was depleted after 30 min. The formation of OH-solanidine (*m/z* 414) was observed in both CYP2D6 and HLM incubations, being faster in CYP2D6. The CL_int_ of solanidine in HLMs was 0.25 mL/min/mg protein and the scaled hepatic clearance 66 L/h, which is 68% of hepatic blood flow (Additional file 1: Table S7).

In HLMs, the CYP2D6-specific inhibitor paroxetine [32] reduced the CL_int_ of 10 nM and 0.1 µM solanidine by 95% and 84%, respectively (Fig. 5c–f). No inhibition was observed with the CYP3A4/5-specific inhibitor ritonavir [33] (Fig. 5c,d). Paroxetine also strongly inhibited the formation of OH-solanidine (*m/z* 414). In addition to OH-solanidine (*m/z* 414), several other solanidine metabolites were formed in the 150 min HLM incubations with a higher solanidine and protein concentration (Additional file 1: Fig. S1). The formation of these metabolites was inhibited by either paroxetine or ritonavir. Incubations with cofactors uridine diphosphate glucuronic acid (UDPGA) in HLM and adenosine 3-phosphate 5′-phosphosulfate triethylammonium salt (PAPS) in HLC fractions showed very minor peaks of solanidine glucuronide and solanidine sulfate, respectively (Additional file 1: Fig. S2). The depletion of solanidine in these incubations was approximately 25% in 30 min.

## Discussion

In this study, the plasma solanidine levels and solanidine metabolites sensitively and specifically identify CYP2D6 activity in humans. In agreement with previous studies [9–13], our data from in vitro and in vivo studies underlines the central role of CYP2D6 enzyme in solanidine metabolism. Furthermore, several solanidine metabolites were detected, and their plasma concentrations and metabolite to solanidine concentration ratios corresponded to the CYP2D6 metabolizer classes of study subjects.

In our study group of 356 healthy volunteers, the fasting plasma concentrations of solanidine varied between < 0.01 and 9.55 ng/mL. Compared to subjects with the CYP2D6 gNM phenotype, the levels of solanidine were nearly 20-fold higher among subjects with the CYP2D6 gPM phenotype. Assuming that the differences in solanidine concentrations between these phenotypes are due to differences in clearances, one could estimate that the fraction of CYP2D6-mediated clearance among individuals with the CYP2D6 gNM phenotype is approximately 95% for solanidine. In addition, the levels of solanidine metabolites *m/z* 414, 416, 444, 430, and 412 were lower in the gPM group than in the gNM group, supporting the previous findings in psychiatric patients [12]. Interestingly, unlike the *m/z* 444 and 430 metabolites, the *m/z* 414 and 412 metabolites showed little difference between the gNM and gUM groups. Moreover, their differences between the gNM and gIM groups were also smaller than those of *m/z* 444 and 430. Therefore, it is tempting to suggest that the *m/z* 444 and 430 metabolites would arise from a further CYP2D6-mediated metabolism of the *m/z* 414 and 412 metabolites, as recently suggested for the formation of *m/z* 444 [11]. Nonetheless, these data strongly support the suggested CYP2D6-mediated biotransformation of solanidine into these metabolites [10, 11].

To elucidate the performance of different metabolites in detecting CYP2D6 activity, we compared the AUPRC and AUROC data for the metabolite to solanidine ratios with solanidine alone. AUPRC expresses the trade-off between precision (positive predictive value) and recall (sensitivity) across different decision thresholds, whereas AUROC shows the relationship between sensitivity and specificity. Of these performance metrics, AUPRC is more useful for imbalanced data sets, in which AUROC could result in too optimistic values. In the present study, both the AUPRC and AUROC analyses showed that solanidine and especially the metabolite to solanidine ratios very effectively differentiated the gPM phenotype from the gNM phenotype. For the gIM and gUM phenotypes, however, solanidine and the ratios performed less well, albeit the supposed secondary metabolites *m*/*z* 444 and 430 to solanidine ratios showed better performance than solanidine alone.

The elimination kinetics of solanidine in humans plays an important role from a biomarker perspective. Although the hepatic clearance of solanidine was predicted to be relatively high, the elimination half-life of solanidine in plasma has been estimated to range from several days to weeks. This long-term presence suggests solanidine accumulates into cells and tissues and has a large volume of distribution [39, 40]. The long elimination half-life may lead to markedly decreased sensitivity in capturing acute CYP2D6 inhibition. The metabolites of solanidine, on the other hand, are more hydrophilic and likely to have smaller distribution volumes and shorter elimination half-lives, and therefore the metabolite to parent solanidine concentration ratios could serve as more responsive CYP2D6 biomarkers than solanidine alone [10]. In a previous study, a one-week treatment with daily dose of a strong CYP2D6 inhibitor paroxetine induced approximately 4.5-fold increase in solanidine concentrations and a significant decrease of most solanidine metabolites [10]. In our study, the metabolite to solanidine concentration ratios for the ions *m/z* 444 and 430, in particular, showed high efficiencies in detecting the different CYP2D6 phenotypes, demonstrating markedly better performance compared to parent solanidine or the metabolites alone. Taken together, our data indicate that the *m/z* 444 and 430 metabolite to solanidine concentration ratios are among the most promising candidate biomarkers for CYP2D6 activity.

Because no authentic reference compounds for solanidine metabolites were commercially available, we compared our analytical data to previous publications [10, 11]. The *m/z* 444 metabolite appears to be the only solanidine metabolite with an identified structure (3,4-*seco*-solanidine-3,4-dioic acid) [11]. MS/MS fragmentation experiments for the *m/z* 444 ion found in our study confirmed a matching pattern of major fragments with the previously published study [11], indicating the same metabolite structure. In our chromatographic system, the analytes were originally separated using a longer analytical column and different mobile phase than in a previously published study [10]. Using an identical analytical method [10], the metabolites in our plasma samples showed equal retention times (rt) to those previously reported for the features *m/z* 414 (rt 4.46 min), 416 (rt 3.88 min), 444 (rt 4.07 min) and 412 (rt 4.22 min), but not for the feature *m/z* 430 (rt 4.14 min vs. 3.85 min) (Additional file 1: Fig. S3). The metabolite *m*/*z* 430 found in our study showed almost identical associations with the *CYP2D6* genotype as *m*/*z* 444, suggesting that they might originate from the same intermediate metabolite. Overall, these data indicate that, except for *m/z* 430, our findings in plasma refer to the same solanidine metabolites as previously described, that is, *m/z* 414.3366, 416.3159, 444.3108, and 412.3210 [10].

In addition to analyses focusing on *CYP2D6* and solanidine, we used two hypothesis free approaches in our study. A non-targeted metabolomics study suggested a strong association of solanidine with the CYP2D6 phenotypes, with no major associations with other metabolites found in human plasma. In a genome-wide association study, we showed that several SNVs in close proximity to the *CYP2D6* gene associate strongly with plasma solanidine metabolite to solanidine ratios. The associated SNVs showed strong linkage disequilibrium with a *CYP2D6* SNV, which defines the no function CYP2D6*4 allele [2]. The lack of other genome-wide significant signals further supports the crucial role of CYP2D6 in solanidine metabolism.

Recently, the *NFIB* gene was found to regulate the activity of several pharmacogenes, including *CYP2D6* [38]. Moreover, individuals carrying the *NFIB* rs28379954 *T* > *C* variant showed increased hydroxylation rate of the CYP2D6 substrate risperidone [38, 41]. In particular, individuals with a *CYP2D6* gNM genotype appeared to convert into gUM if they also carried the *NFIB* variant*.* Therefore, we genotyped all study subjects for *NFIB* rs28379954. In our study, however, carrying the rs28379954 *T* > *C* variant did not appear to convert CYP2D6 gNMs to gUMs.

To further validate our findings, we performed a series of in vitro experiments with human recombinant CYP enzymes, and with HLM fractions. Among the 11 studied CYP enzymes, solanidine was mainly metabolized by CYP2D6, with minor contribution from CYP3A4. Furthermore, inhibition of CYP2D6 in HLMs nearly completely abolished the metabolism of solanidine, especially when investigated at a clinically relevant solanidine concentration. In addition, only moderate depletion of solanidine and trace amounts of solanidine glucuronide and solanidine sulfate were detected in HLM and HLC incubations containing the UGT-related cofactor UDPGA or the SULT-related cofactor PAPS, respectively. This suggests that glucuronidation or sulfation are not of major importance for solanidine elimination. These in vitro observations also support that CYP2D6 is the major enzyme in the metabolism of solanidine.

CYP enzymes have assumedly evolved by so called plant-animal warfare since animals began to ingest plants. In response, plants developed toxic secondary metabolites such as alkaloids, terpenes, or tannins to fight against herbivores and pathogens. Selective pressure for dietary detoxification resulted in the expansion of CYP enzymes to detoxify these compounds and allowed usage of further plant species, and CYP2D6 is known for its high affinity to plant toxins, including alkaloids [42–44]. Potato (*Solanum tuberosum*) is nowadays cultivated in more than 160 countries worldwide. It is the third most important food crop after rice and wheat globally [46] which makes it the main source of solanidine for humans. The main glycoalkaloids of potato, α-solanine and α-chaconine, form approximately 95% of total glycoalkaloids of commercially available potatoes. Solanidine is the aglycone form of these two, derived by carbohydrate site removal by hydrolysis [47–49].

The ability to quantify solanidine and its metabolites in human plasma depends on the intake of alkaloid containing plants, such as potatoes and tomatoes. In our study, 41 study subjects did not have detectable solanidine concentrations in their plasma samples, suggesting that these individuals had not ingested solanine-containing products recently. Therefore, our study is limited by the variation in potato intake among participants. To address this, future studies should employ standardized assessment of intake of solanine-containing products to more comprehensively assess solanidine’s role as a biomarker for CYP2D6 activity. It is noteworthy that due to the apparent long half-life of solanidine, it is expected to be present in plasma over a relatively long time. Without knowledge on solanine intake, however, our study successfully demonstrated a 40-fold difference in plasma solanidine concentration between the extreme metabolic classes, i.e., the gPM and gUM phenotypes. This indicates that when solanidine is quantifiable in plasma, its fasting concentration is strongly dependent on CYP2D6 activity. Although our GWAS analyses showed strong associations between the metabolite-to-solanidine ratios and the CYP2D6 locus, minor genetic influences of other loci cannot be ruled out due to the limited sample size. It should also be noted that our in vitro solanidine sulfation and glucuronidation experiments did not include positive controls, and they should therefore be considered preliminary.

The known toxicity of potato alkaloids must be taken into account when considering potato as a food-derived CYP2D6 probe substrate. In our study, the concentrations of solanidine did not exceed safe levels even among poor CYP2D6 metabolizers who had nearly 20-fold higher solanidine concentrations compared to normal metabolizers [47, 50]. Normal usage of potato in the diet does not elevate the plasma alkaloid concentrations to hazardous levels even with possible strong CYP2D6 inhibition. In addition, the ability of detecting solanidine in humans with low concentrations and its long half-time further supports its benefits as a dietary biomarker. Green potatoes, however, should not be ingested due to higher solanidine concentrations and possible risk for toxicity [47, 48].

## Conclusions

In summary, plasma solanidine and solanidine metabolite to solanidine ratios were highly efficient in detecting CYP2D6 gPM, gIM, gNM, and gUM phenotypes among healthy volunteers, suggesting a high potential for quantifying CYP2D6 activity in addition to genotyping methods. Further studies are warranted to elucidate the structures of the unknown solanidine metabolites. Moreover, evaluation of potato intake is needed to assess an appropriate baseline plasma solanidine level for its optimal use as a CYP2D6 biomarker.

### Supplementary Information

**Additional file 1**. **Figure S1**. Effects of CYP2D6 and CYP3A inhibition on the formation of solanidine metabolites. The effects of paroxetine (CYP2D6 inhibitor) and ritonavir (CYP3A inhibitor) on the formation of various solanidine metabolites, including OH-solanidine (m/z 414/98, rt 6.5 min, marked with a star*), from solanidine 5 µM were investigated in HLMs (2 mg/ml) for up to 150 min. CYP, cytochrome P450; HLMs, human liver microsomes. **Figure S2**. Phase II metabolism of solanidine. UGT-mediated metabolism of solanidine was investigated by incubation of solanidine (0.1 µM) with HLMs (0.2 mg/ml) and UDPGA (a-b) for 30 min, and SULT-mediated metabolism by incubation of solanidine (0.1 µM) with HLC (0.1 mg/ml) and PAPS (c-d) for 30 min. HLC, human liver cytosol; HLMs, human liver microsomes; PAPS, adenosine 3-phosphate 5′-phosphosulfate triethylammonium salt; SULT, sulfotransferase; UDPGA, uridine 5′-diphospho-glucuronic acid; UGT, uridine diphospho-glucuronosyltransferase. **Figure S3**. Chromatograms of solanidine and its selected CYP2D6 mediated metabolites in plasma. a) Separation of compounds using the chromatographic settings described by Magliocco et al. 2021 and b) using the method presented in this study. **Table S1**. Demographic characteristics of study subjects. **Table S2**. CYP2D6 star allele definitions. **Table S3**. CYP2D6 genotype distribution in the study population. **Table S4**. Associations of metabolite features found in non-targeted metabolomics analysis of human plasma with CYP2D6 phenotypes. **Table S5**. Effect of CYP2D6 phenotype on plasma solanidine metabolites in healthy volunteers (n=314). **Table S6**. Effect of NFIB adjusted CYP2D6 phenotype on plasma solanidine and metabolite to solanidine ratios in healthy volunteers (n=314). **Table S7**. Solanidine clearance values.

## Data Availability

The datasets used and/or analyzed during the current study are available to the extent allowed by the informed consent and the EU General Data Protection Regulation and other applicable regulation from the corresponding author on reasonable request.

## Abbreviations

AS: Activity score
AUPRC: Area under the precision–recall curve
AUROC: Area under the receiver operating characteristic curve
CLint: Intrinsic clearance
CPIC: The clinical pharmacogenetics implementation consortium
CV: Coefficients of variation
CYP: Cytochrome P450
DDI: Drug–drug interaction
EDTA: Ethylenediaminetetraacetic acid
ESI: Electrospray ionization
GBDT: Gradient boosted decision trees
gIM: Genetically intermediate metabolizer
gNM: Genetically normal metabolizer
gUM: Genetically ultrarapid metabolizer
gPM: Genetically poor metabolizer
HILIC: Hydrophilic interaction chromatography
HLC: Human liver cytosol
HLM: Human liver microsome
IS: Internal standard
LLOQ: Lower limit of quantification
*m*/*z*: Mass-to-charge ratio
NADPH: Nicotinamide adenine dinucleotide phosphate
PAPS: Adenosine 3-phosphate 5′-phosphosulfate triethylammonium salt
PharmVar: PHARMACOGENE variation consortium
PR: Precision recall analysis
RF: Random forest
ROC: Receiver operating characteristic
RP: Reversed phase
SD: Standard deviation
*S*/*N*: Signal-to-noise
SNV: Single nucleotide variation
SSDA: 3,4-Seco-solanidine-3,4-dioic acid
SULT: Sulfotransferase
TUDCA: Tauroursodeoxycholate
UDPGA: Uridine diphosphate glucuronic acid
UGT: Diphospho-glucuronosyltransferase
UHPLC: Ultra-high-performance liquid chromatography
