# Supplementary material for: Solanidine is a sensitive and specific dietary biomarker for CYP2D6 activity
Source: Hum Genomics. 2024 Feb 1;18:11. doi: 10.1186/s40246-024-00579-8 (PMC10835938; doi:10.1186/s40246-024-00579-8)
Supplement: Supplementary file 1 — Additional file 1. Figure S1. Effects of CYP2D6 and CYP3A inhibition on the formation of solanidine metabolites. The effects of paroxetine (CYP2D6 inhibitor) and ritonavir (CYP3A inhibitor) on the formation of various solanidine metabolites, including OH-solanidine (m/z 414/98, rt 6.5 min, marked with a star*), from solanidine 5 µM were investigated in HLMs (2 mg/ml) for up to 150 min. CYP, cytochrome P450; HLMs, human liver microsomes. Figure S2. Phase II metabolism of solanidine. UGT-mediated metabolism of solanidine was investigated by incubation of solanidine (0.1 µM) with HLMs (0.2 mg/ml) and UDPGA (a-b) for 30 min, and SULT-mediated metabolism by incubation of solanidine (0.1 µM) with HLC (0.1 mg/ml) and PAPS (c-d) for 30 min. HLC, human liver cytosol; HLMs, human liver microsomes; PAPS, adenosine 3-phosphate 5′-phosphosulfate triethylammonium salt; SULT, sulfotransferase; UDPGA, uridine 5′-diphospho-glucuronic acid; UGT, uridine diphospho-glucuronosyltransferase. Figure S3. Chromatograms of solanidine and its selected CYP2D6 mediated metabolites in plasma. a) Separation of compounds using the chromatographic settings described by Magliocco et al. 2021 and b) using the method presented in this study. Table S1. Demographic characteristics of study subjects. Table S2. CYP2D6 star allele definitions. Table S3. CYP2D6 genotype distribution in the study population. Table S4. Associations of metabolite features found in non-targeted metabolomics analysis of human plasma with CYP2D6 phenotypes. Table S5. Effect of CYP2D6 phenotype on plasma solanidine metabolites in healthy volunteers (n=314). Table S6. Effect of NFIB adjusted CYP2D6 phenotype on plasma solanidine and metabolite to solanidine ratios in healthy volunteers (n=314). Table S7. Solanidine clearance values. [file 40246_2024_579_MOESM1_ESM.pdf]

Supplementary Methods

In vitro studies

Pooled human liver microsomes (HLM) and human liver cytosol fraction (HLC) (both mixed gender, pool of 200) were purchased from Sekisui Xenotech (Kansas City, MO). Human recombinant CYP enzyme Bactosomes expressed in *Escherichia coli* were purchased from Cypex (Dundee, Scotland, UK). Solanidine was obtained from Phytolab GmbH & co (Vestenbergsgreuth, Germany). Paroxetine hydrochloride and ritonavir were purchased from Toronto Research Chemicals (Toronto, ON, Canada), adenosine 3-phosphate 5′-phosphosulfate triethylammonium salt (PAPS), nicotinamide adenine dinucleotide phosphate (NADPH), and uridine 5′-diphospho-glucuronic acid (UDPGA) from Sigma-Aldrich (St Louis, MO), and alamethicin from Cayman Chemical (Ann Arbor, MI). All other chemicals and solvents were of standard analytical grade. The stock solutions of solanidine were prepared in methanol.

All *in vitro* incubations were carried out in phosphate buffer (100 mM, pH 7.4) as triplicates in polypropylene microcentrifuge tubes on a heated shaker at 37°C, 300 rpm (Thermomixer C, Eppendorf, Hamburg, Germany). Reactions were initiated by addition of solanidine to the reaction mixtures and stopped at indicated time points by transfer of 50 µL mixture into tubes containing 150 µL ice-cold methanol and isotope labeled tauroursodeoxycholate (TUDCA-D5; internal standard). The samples were centrifuged for 15 min at 21,000 *g*. Thereafter, supernatants (150 µL) were transferred to liquid chromatography microvials and diluted with H_2O (300 µL) for liquid chromatography-tandem mass spectrometry analysis. All incubations contained ≤ 1% methanol.

First, the formation of hydroxy (OH) solanidine (*m/z* 414) from solanidine was screened in human recombinant CYP1A2, CYP2A6, CYP2B6, CYP2C8, CYP2C9, CYP2C19, CYP2D6, CYP2E1,

CYP2J2, CYP3A4, CYP3A5, and control Bactosomes. Each reaction mixture contained NADPH (1 mM), solanidine (2 µM), and Bactosomes (0.1 mg/mL total protein). Preincubation time was 5 min and samples for analyses were taken at 0, 20, and 40 min.

Then, the metabolic depletion of a low, clinically relevant concentration of solanidine (10 nM) was investigated in HLMs (0.2 mg/mL protein) and human recombinant CYP2D6 (0.1 mg/mL Bactosome protein) in the presence of NADPH (1 mM). Preincubation time was 5 min, and samples were taken at 0, 5, 15, 30, and 60 min.

Inhibition experiments were carried out in HLMs using paroxetine as a mechanism-based inhibitor of CYP2D6 and ritonavir as a mechanism-based inhibitor of CYP3A4/5.[1,2] Due to time-dependency of the mechanism-based inhibition, paroxetine (1 µM) or ritonavir (0.1 µM) were first pre-incubated with HLMs (0.2 mg/mL) and NADPH (1 mM) for 30 min prior to addition of solanidine (0.1 µM) to the reaction mixtures. The inhibition by paroxetine was also investigated with a low solanidine concentration of 10 nM. Control samples were done without the inhibitors. Samples were taken at 0, 5, 15, 30, and 60 min.

To investigate the formation of solanidine metabolites other than OH-solanidine (*m/z* 414), additional experiments with higher concentrations of HLM (2 mg/mL) and solanidine (5 µM), and a longer incubation time (150 min) were carried out. Here, the NADPH RapidStart Regeneration System for Extended Metabolism (Xenotech) was used to regenerate NADPH to the reaction. These incubations were also done with and without paroxetine (1 µM) and ritonavir (0.1 µM).

To investigate the possible contribution of uridine diphospho-glucuronosyltransferase (UGT) and sulfotransferase (SULT) enzymes to solanidine metabolism, solanidine was incubated in HLMs with

UGT-related cofactor UDPGA and in HLC fraction with SULT-related cofactor PAPS. The reaction mixtures contained either 1) HLM (0.2 mg/mL protein), pore-forming peptide alamethicin (10 µg/mL), UDPGA (5 mM), $MgCl_2$ (10 mM), and solanidine (0.1 µM), or 2) HLC (0.1 mg/mL protein), PAPS (100 µM), $MgCl_2$ (10 mM), and solanidine (0.1 µM). Preincubation time was 5 min and samples were taken at 0 and 30 min.

The kinetics of solanidine depletion in the HLM depletion and inhibition experiments were analyzed using GraphPad Prism (version 7.03; GraphPad Software, Inc., San Diego, CA, USA). Depletion rate constants (k_{dep}) were determined using linear regression of ln-transformed solanidine concentrations, and the intrinsic clearance (CL_{int}) of solanidine was expressed as $CL_{int} = k_{dep}/[M]$, where [M] is the HLM protein concentration used in the experiments (0.2 mg/mL). The change in solanidine CL_{int} due to inhibition was calculated by comparing the CL_{int} values in samples containing the inhibitors with the CL_{int} value in the control samples.

All CL_{int} values were corrected for non-specific binding to protein by $CL_{int,u} = CL_{int}/f_{u,mic}$, where $CL_{int,u}$ is the unbound intrinsic clearance and $f_{u,mic}$ is the predicted unbound fraction of drug (0.377) in the incubations (https://members.simcyp.com/account/tools/fumic/, accessed on Feb 9, 2023). Measured HLM $CL_{int,u}$ values were scaled to $CL_{int,\textit{in vivo}}$ using 39.79 mg microsomal protein/g liver, and liver volume and density values of 1.65 l and 1,080 g/l liver (Simcyp Population-Based Simulator V20). In the final step, hepatic blood clearance (CL_H) values were calculated using the well-stirred model.[3]

$$CL_H = Q_H \times \frac{f_{u,B} \times CL_{int,in\ vivo}}{Q_H + f_{u,B} \times CL_{int,in\ vivo}}$$

where Q_H is the hepatic blood flow (1.61 l/min) [4] and $f_{u,B}$ is the unbound fraction of solanidine in blood. $f_{u,B}$ was calculated according to $f_{u,B} = f_{u,p} \times 1/BP$, where $f_{u,p}$ and BP are the predicted

unbound fraction in plasma (0.065) and blood-to-plasma concentration ratio (0.862) of solanidine, respectively (https://members.simcyp.com/account/tools/fumic/, accessed on Feb 9, 2023). The calculated $f_{u,B}$ equaled to 0.075.

References:

1. Bertelsen, K.M., Venkatakrishnan, K., Von Moltke, L.L., Obach, R.S., Greenblatt, D.J. Apparent mechanism-based inhibition of human CYP2D6 in vitro by paroxetine: comparison with fluoxetine and quinidine. Drug Metab. Dispos. 2003;31:289-293.
2. Koudriakova, T., Iatsimirskaia, E., Utkin, I., Gangl, E., Vouros, P., Storozhuk, E., et al. Metabolism of the human immunodeficiency virus protease inhibitors indinavir and ritonavir by human intestinal microsomes and expressed cytochrome P4503A4/3A5: mechanism-based inactivation of cytochrome P4503A by ritonavir. Drug Metab. Dispos. 1998;26:552-561.
3. Yang, J., Jamei, M., Yeo, K.R., Rostami-Hodjegan, A., Tucker, G.T. Misuse of the well-stirred model of hepatic drug clearance. *Drug Metab Dispos*. 2007;**35**:501-2.
4. Pelkonen, O., Turpeinen, M. In vitro-in vivo extrapolation of hepatic clearance: biological tools, scaling factors, model assumptions and correct concentrations. *Xenobiotica* 2007;**37**: 1066-89.

Table S1. Demographic characteristics of study subjects

CYP2D6 phenotype	Age (years)	Weight (kg)	BMI (kg/m^2)	Sex
poor metabolizer	23.3 ± 2.7	67.2 ± 15.1	23.4 ± 3.5	5 women, 4 men
intermediate metabolizer	23.9 ± 4.0	70.8 ± 13.0	22.9 ± 2.9	43 women, 52 men
normal metabolizer	24.1 ± 4.0	69.5 ± 11.7	22.9 ± 2.5	118 women, 112 men
ultrarapid metabolizer	24.2 ± 5.7	67.0 ± 11.9	22.9 ± 3.0	16 women, 5 men
all	24.1 ± 4.1	69.7 ± 12.1	22.9 ± 2.7	183 women, 173 men

Data are mean ± standard deviation.

Table S2. CYP2D6 star allele definitions.

PharmVar *allele	Sequence variation	dbSNP ID
**2*	2851C>T, 4181G>C	rs16947, rs1135840
**3*	2550delA	rs35742686
**4*	1847G>A	rs3892097
**5*	full gene deletion	
**6*	449A>C, 1708delT	rs867984132, rs5030655
**9*	2616delAAG	rs5030656
**10*	100C>T, 4181G>C	rs1065852, rs1135840
**11*	882G>C, 2851C>T, 4181G>C	rs201377835, rs16947, rs1135840
**13*	CYP2D7-CYP2D6 hybrid gene	
**17*	1022C>T, 2851C>T, 4181G>C	rs28371706, rs16947, rs1135840
**29*	1660G>A+1662G>C, 2851C>T, 3184G>A, 4181G>C	rs61736512 + rs1058164, rs16947, rs59421388, rs1135840
**39*	4181G>C	rs1135840
**41*	851C>T, 2989G>A, 4181G>C	rs16947, rs28371725, rs1135840
**59*	2851C>T, 2940G>A, 4181G>C	rs16947, rs79292917, rs1135840
**65*	100C>T, 2851C>T, 4181G>C	rs1065852, rs16947, rs1135840,
**69*	100C>T, 2851C>T, 2989G>A, 4181G>C	rs1065852, rs16947, rs28371725, rs1135840
**88*	1013T>C, 4181G>C	rs76187628, rs1135840

The HGVS reference to all *CYP2D6* core alleles is NG_008376.4 and all sequence variations are named from ATG start as per PharmVar guidelines.

Table S3. *CYP2D6* genotype distribution in the study population of 356 healthy Finnish volunteers.

Predicted phenotype (%)	Activity score	Genotype (%)
PM (2.5%)	0	**3/*4* (0.8%), **4/*4* (1.1%), **4/*5* (0.3%), **4/*6* (0.3%)
IM (26.7%)	0.25-1	**1/*3* (1.7%), **1/*4* (6.5%), **1/*5* (1.7%), **1/*6* (0.3%), **2/*3* (2.8%), **2/*4* (8.1%), **2/*5* (1.1%), **2/*6* (0.3%), **3/*41* (0.6%), **4/*17* (0.3%), **4/*41* (2.2%), **4/*9* (0.3%), **5/*41* (0.3%), **6/*10* (0.3%), **6/*41* (0.3%)
NM (64.6 %)	1.25-2.25	**1/*1* (16.6%), **1/*2* (26.7%), **1/*9* (1.1%), **1/*10* (1.4%), **1/*41* (3.4%), **1/*59* (0.8%), **1x2/*4* (0.6%), **2/*2* (7.6%), **2/*10* (2%), **2/*41* (1.7%), **2*/59* (1.1%), **2*/9* (1.1%), **2x2/*4* (0.3%), **2x2/*41* (0.3%)
UM (5.9%)	>2.25	**1/*1x2* (1.7%), **1/*2x2* (1.4%), **1x2/*2* (0.8%), **2/*2x2* (2%)
NA (0.3%)	NA	NA (0.3%)

IM, intermediate metabolizer; NA, undetermined; NM, normal metabolizer, PM, poor metabolizer; UM, ultrarapid metabolizer

Table S4. Associations of metabolite features found in non-targeted metabolomics analysis of human plasma with CYP2D6 phenotypes

GINI impurity decrease							
**GBDT**	**RF**	**Feature**	**Level of identification**	**Molecular weight**	**Retention time [min]**	**Chromatography**	**Ionization mode**
0.166355	0.049395	Solanidine	LI 1	397.33447	0.53	HILIC	Positive
0.038309	0.111721	PE group lipid	LI 3	477.29156	0.65	HILIC	Positive
0.024206	0.012204		LI 4	168.03856	7.29	HILIC	Positive
0.023565			LI 4	192.09012	0.89	HILIC	Positive
0.022470			LI 4	336.05673	0.69	RP	Negative
0.018008			LI 4	521.33869	1.12	HILIC	Positive
0.016571			LI 4	63.00941	4.20	HILIC	Positive
0.015283			LI 4	200.85681	15.52	RP	Negative
0.014896			LI 4	297.62827	10.26	RP	Positive
0.014861	0.010449		LI 4	313.20995	0.50	HILIC	Positive
0.012943			LI 4	565.88142	4.19	HILIC	Positive
0.012656			LI 4	216.03107	15.46	RP	Positive
0.012432		Piperine	LI 2	285.13638	8.40	RP	Positive
0.011873			LI 4	557.30469	0.45	HILIC	Positive
0.011557			LI 4	298.25072	11.40	RP	Positive
0.011384			LI 4	521.33869	1.12	RP	Positive
0.011103			LI 4	314.85835	0.47	HILIC	Negative
0.010257		cyclo(delta-Ala-L-Val)	LI 3	168.08993	2.29	RP	Positive
0.010143			LI 4	186.13689	4.54	HILIC	Positive

HILIC, hydrophilic interaction liquid chromatography; LI, level of identification; PE, phosphatidylethanolamine; RP, reversed phase liquid chromatography

Table S5. Effect of CYP2D6 phenotype on plasma solanidine metabolites in healthy volunteers (n=314)

Phenotype (n)	Geometric mean (95% CI)	Ratio to normal function (95% CI)	*P*
**Feature: m/z 414**			
Poor metabolizer (9)	0.0115 (0.00548, 0.0239)	0.0171 (0.00807, 0.0365)	1.34×10^{-22}
Intermediate metabolizer (89)	0.848 (0.671, 1.07)	1.27 (0.958, 1.68)	0.0970
Normal metabolizer (196)	0.668 (0.571, 0.782)	1.00	
Ultra rapid metabolizer (20)	0.419 (0.254, 0.689)	0.627 (0.372, 1.06)	0.0795
**Feature: m/z 416**			
Poor metabolizer (9)	0.00764 (0.00396, 0.0147)	0.0152 (0.00776, 0.0298)	2.51×10^{-28}
Intermediate metabolizer (89)	0.476 (0.386, 0.586)	0.947 (0.736, 1.22)	0.669
Normal metabolizer (196)	0.503 (0.437, 0.578)	1.00	
Ultra rapid metabolizer (20)	0.464 (0.298, 0.724)	0.924 (0.580, 1.47)	0.740
**Feature: m/z 444**			
Poor metabolizer (9)	0.00563 (0.00299, 0.0106)	0.0557 (0.0291, 0.107)	1.52×10^{-16}
Intermediate metabolizer (89)	0.0591 (0.0483, 0.0722)	0.583 (0.458, 0.744)	1.74×10^{-5}
Normal metabolizer (196)	0.101 (0.0884, 0.116)	1.00	
Ultra rapid metabolizer (20)	0.158 (0.103, 0.242)	1.56 (0.993, 2.44)	0.0538
**Feature: m/z 430**			
Poor metabolizer (9)	0.00743 (0.00370, 0.0149)	0.0809 (0.0396, 0.165)	2.34×10^{-11}
Intermediate metabolizer (89)	0.0537 (0.0430, 0.0669)	0.583 (0.447, 0.762)	8.67×10^{-5}
Normal metabolizer (196)	0.0920 (0.0792, 0.107)	1.00	
Ultra rapid metabolizer (20)	0.148 (0.0928, 0.238)	1.61 (0.985, 2.64)	0.0572
**Feature: m/z 412**			
Poor metabolizer (9)	0.00584 (0.00281, 0.0121)	0.278 (0.132, 0.586)	8.38×10^{-4}
Intermediate metabolizer (89)	0.0235 (0.0187, 0.0296)	1.12 (0.846, 1.48)	0.429
Normal metabolizer (196)	0.0210 (0.0180, 0.0246)	1.00	
Ultra rapid metabolizer (20)	0.0201 (0.0123, 0.0329)	0.956 (0.570, 1.60)	0.863

Metabolite concentrations are given in arbitrary units relative to the metabolite to internal standard peak area ratio.

Table S6. Effect of NFIB adjusted CYP2D6 phenotype on plasma solanidine and metabolite to solanidine ratios in healthy volunteers (n=314)

**Phenotype (n)**	**Geometric mean (95% CI)**	**Ratio to normal function (95% CI)**	***P***
**Feature: Solanidine (ng/mL)**			
Poor metabolizer (9)	1.77 (0.775, 4.04)	19.9 (8.55, 46.5)	2.10×10^{-11}
Intermediate metabolizer (89)	0.154 (0.119, 0.200)	1.74 (1.26, 2.39)	7.75×10^{-4}
Normal metabolizer (179)	0.0887 (0.0738, 0.107)	1.00	
Ultrarapid metabolizer (37)	0.0711 (0.0474, 0.107)	0.801 (0.513, 1.25)	0.329
**Feature: m/z 414 to solanidine peak area ratio**			
Poor metabolizer (9)	0.00649 (0.00441, 0.00953)	0.000850 (0.000573, 0.00126)	8.33×10^{-110}
Intermediate metabolizer (89)	5.49 (4.86, 6.20)	0.720 (0.620, 0.836)	2.06×10^{-5}
Normal metabolizer (179)	7.63 (7.00, 8.31)	1.00	
Ultrarapid metabolizer (37)	6.82 (5.64, 8.25)	0.895 (0.726, 1.10)	0.294
**Feature: m/z 416 to solanidine peak area ratio**			
Poor metabolizer (9)	0.00432 (0.00230, 0.00811)	0.000759 (0.000398, 0.00145)	1.29×10^{-64}
Intermediate metabolizer (89)	3.08 (2.53, 3.77)	0.542 (0.424, 0.692)	1.33×10^{-6}
Normal metabolizer (179)	5.69 (4.95, 6.55)	1.00	
Ultrarapid metabolizer (37)	6.59 (4.83, 8.99)	1.16 (0.823, 1.63)	0.400
**Feature: m/z 444 to solanidine peak area ratio**			
Poor metabolizer (9)	0.00318 (0.00156, 0.00649)	0.00280 (0.00135, 0.00583)	1.46×10^{-41}
Intermediate metabolizer (89)	0.383 (0.305, 0.480)	0.338 (0.256, 0.445)	1.68×10^{-13}
Normal metabolizer (179)	1.13 (0.967, 1.33)	1.00	
Ultrarapid metabolizer (37)	1.70 (1.19, 2.41)	1.50 (1.02, 2.20)	0.0402
**Feature: m/z 430 to solanidine peak area ratio**			
Poor metabolizer (9)	0.00419 (0.00214, 0.00817)	0.00406 (0.00205, 0.00805)	1.20×10^{-41}
Intermediate metabolizer (89)	0.347 (0.281, 0.430)	0.337 (0.260, 0.436)	4.24×10^{-15}
Normal metabolizer (179)	1.03 (0.889, 1.20)	1.00	
Ultrarapid metabolizer (37)	1.55 (1.12, 2.16)	1.51 (1.05, 2.16)	0.0267
**Feature: m/z 412 to solanidine peak area ratio**			
Poor metabolizer (9)	0.00330 (0.00206, 0.00527)	0.0136 (0.00842, 0.0220)	2.13×10^{-48}
Intermediate metabolizer (89)	0.152 (0.131, 0.177)	0.629 (0.525, 0.755)	9.38×10^{-7}
Normal metabolizer (179)	0.242 (0.218, 0.269)	1.00	
Ultrarapid metabolizer (37)	0.258 (0.205, 0.325)	1.07 (0.827, 1.37)	0.621

Table S7. Solanidine clearance values. Measured *in vitro* and $f_{u,mic}$-corrected CL_{int} values of solanidine depletion (10 nM) in HLM incubations are shown together with the scaled $CL_{int,\textit{in vivo}}$ and calculated hepatic blood clearance of solanidine. Several assumptions were done in the calculations, as described in the methods. Values shown are mean ± standard deviation of triplicates.

CL_{int} (mL/min/mg)	$CL_{int,u}$ (mL/min/mg)[a]	$CL_{int,\textit{in vivo}}$ (L/h)[b]	CL_H (L/h)[c]
0.25 ± 0.03	0.66 ± 0.08	46.9 ± 5.53	66.2 ± 2.43

[a] This value has been corrected for predicted non-specific binding to protein according to $CL_{int,u} = CL_{int}/f_{u,mic}$.
[b] This value was obtained by multiplying HLM $CL_{int,u}$ with scaling factors.
[c] This value was calculated using the well-stirred model.[1]

1. Yang, J., Jamei, M., Yeo, K.R., Rostami-Hodjegan, A., Tucker, G.T. Misuse of the well-stirred model of hepatic drug clearance. *Drug Metab Dispos*. **35**, 501-2 (2007).

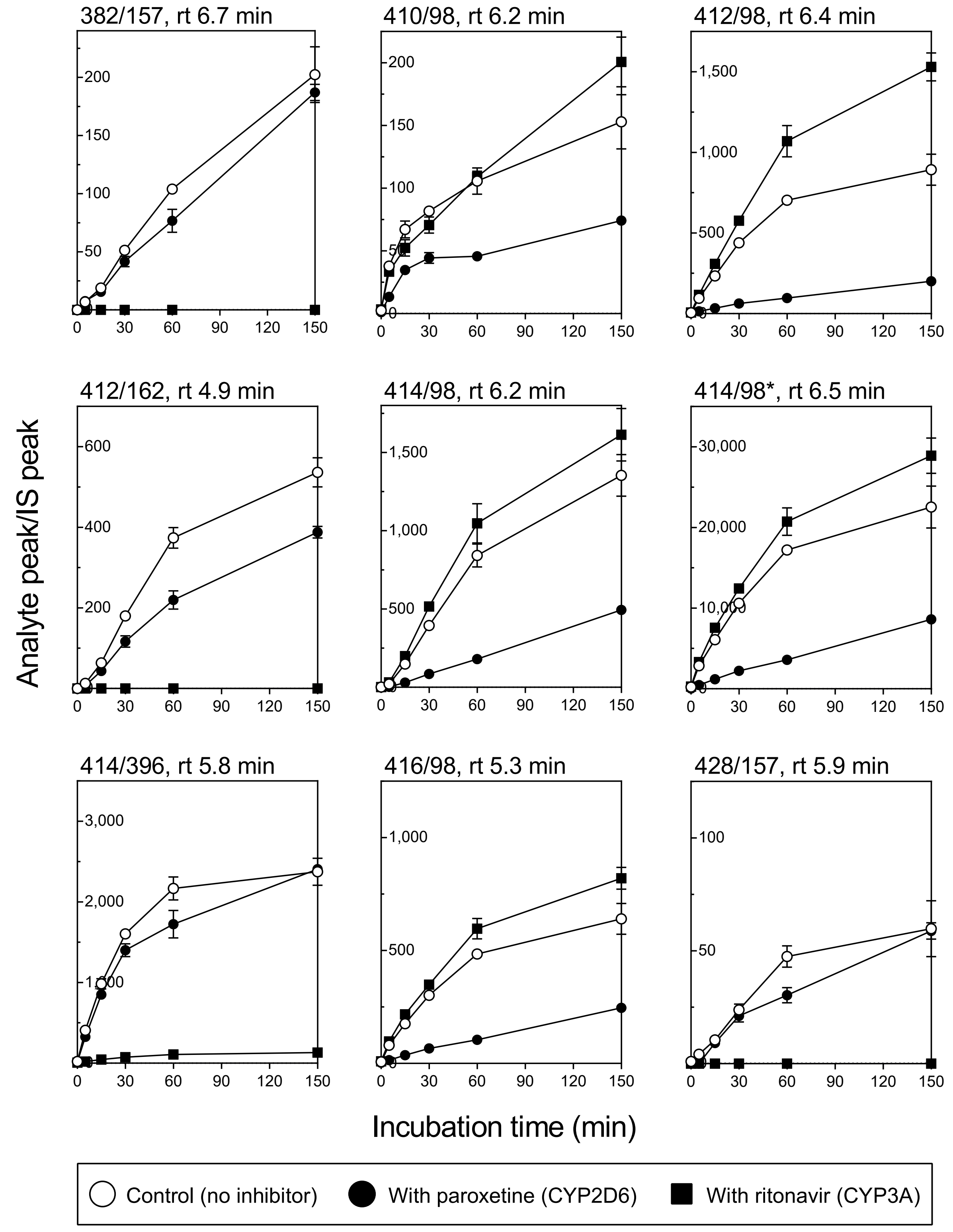

382/157, rt 6.7 min
410/98, rt 6.2 min
412/98, rt 6.4 min
412/162, rt 4.9 min
414/98, rt 6.2 min
414/98*, rt 6.5 min
414/396, rt 5.8 min
416/98, rt 5.3 min
428/157, rt 5.9 min
Analyte peak/IS peak
Incubation time (min)
Control (no inhibitor)
With paroxetine (CYP2D6)
With ritonavir (CYP3A)

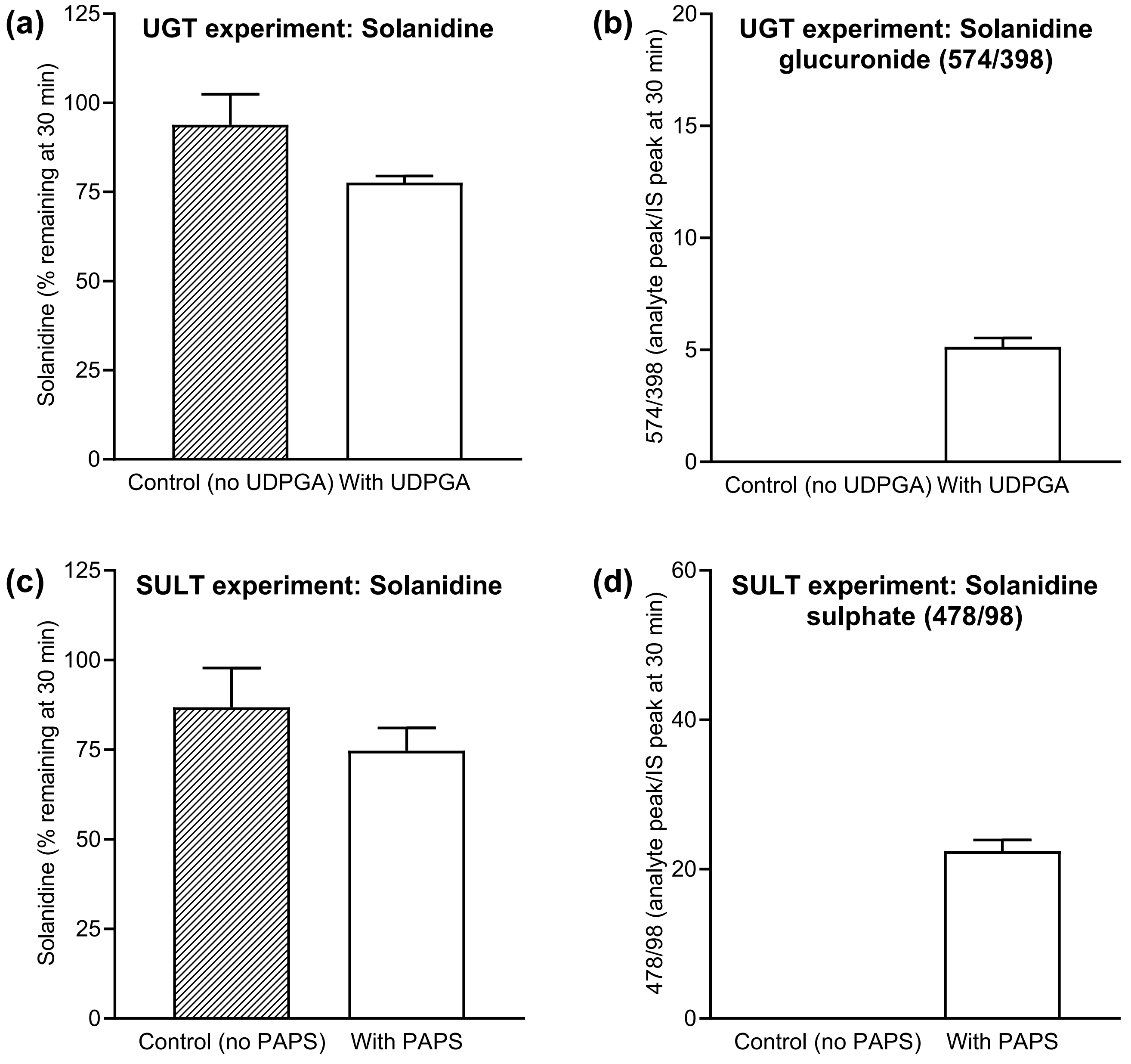

(a)
UGT experiment: Solanidine
Solanidine (% remaining at 30 min)
125
100
75
50
25
0
Control (no UDPGA)
With UDPGA
(b)
UGT experiment: Solanidine glucuronide (574/398)
574/398 (analyte peak/IS peak at 30 min)
20
15
10
5
0
Control (no UDPGA)
With UDPGA
(c)
SULT experiment: Solanidine
Solanidine (% remaining at 30 min)
125
100
75
50
25
0
Control (no PAPS)
With PAPS
(d)
SULT experiment: Solanidine sulphate (478/98)
478/98 (analyte peak/IS peak at 30 min)
60
40
20
0
Control (no PAPS)
With PAPS

a)

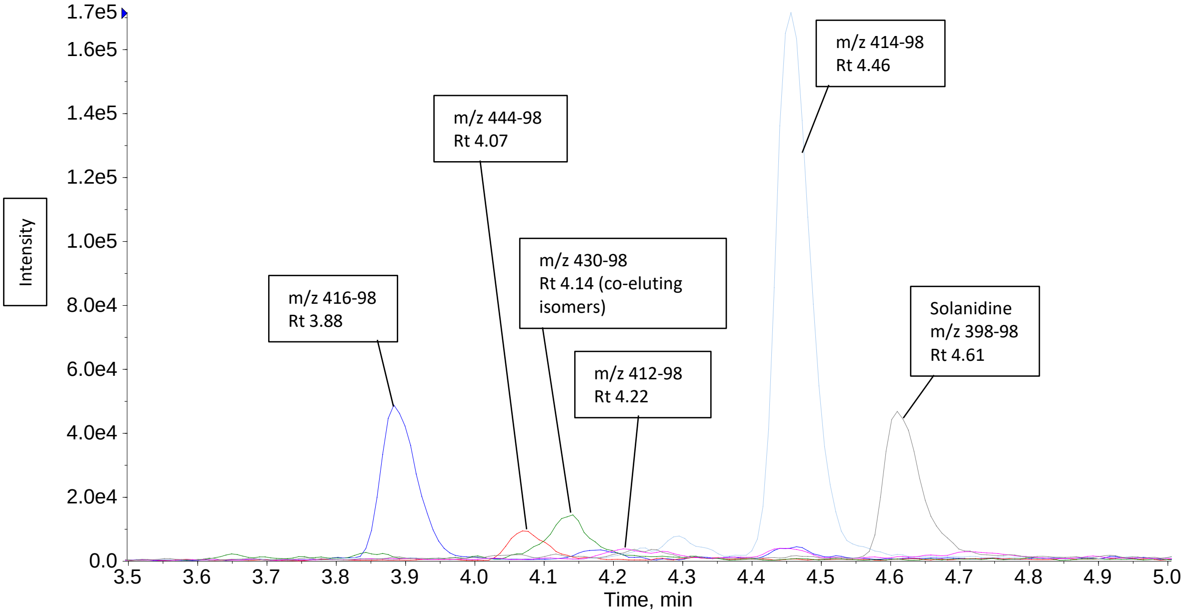
Intensity
1.7e5
1.6e5
1.4e5
1.2e5
1.0e5
8.0e4
6.0e4
4.0e4
2.0e4
0.0
m/z 416-98
Rt 3.88
m/z 444-98
Rt 4.07
m/z 430-98
Rt 4.14 (co-eluting isomers)
m/z 412-98
Rt 4.22
m/z 414-98
Rt 4.46
Solanidine
m/z 398-98
Rt 4.61
3.5 3.6 3.7 3.8 3.9 4.0 4.1 4.2 4.3 4.4 4.5 4.6 4.7 4.8 4.9 5.0
Time, min

b)

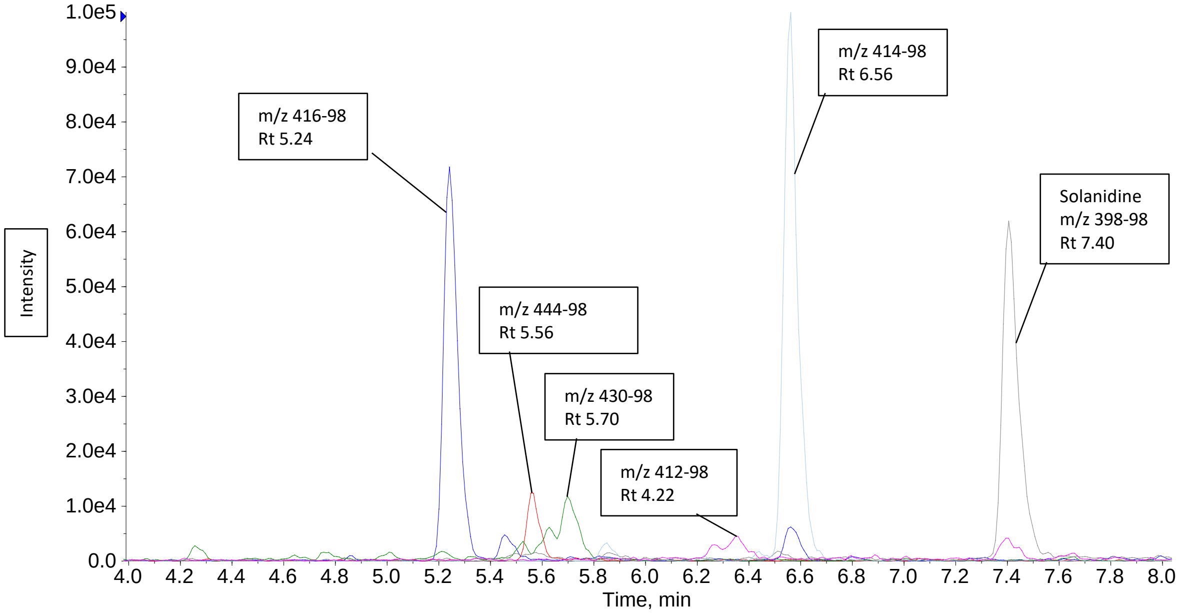
Intensity
1.0e5
9.0e4
8.0e4
7.0e4
6.0e4
5.0e4
4.0e4
3.0e4
2.0e4
1.0e4
0.0
m/z 416-98
Rt 5.24
m/z 444-98
Rt 5.56
m/z 430-98
Rt 5.70
m/z 412-98
Rt 4.22
m/z 414-98
Rt 6.56
Solanidine
m/z 398-98
Rt 7.40
4.0 4.2 4.4 4.6 4.8 5.0 5.2 5.4 5.6 5.8 6.0 6.2 6.4 6.6 6.8 7.0 7.2 7.4 7.6 7.8 8.0
Time, min
